# Corneal Copper Deposition Secondary to Monoclonal Gammopathy in a Patient With Chronic Lymphocytic Leukemia: A Case Report

**DOI:** 10.7759/cureus.50801

**Published:** 2023-12-19

**Authors:** Koichiro Shinji, Tai-ichiro Chikama, Taka-aki Moriguchi, Atsuhiko Fukuto, Yoshiaki Kiuchi

**Affiliations:** 1 Department of Ophthalmology and Visual Science, Graduate School of Biomedical Sciences, Hiroshima University, Hiroshima, JPN; 2 Department of Ophthalmology, Hiroshima Prefectural Hospital, Hiroshima, JPN; 3 Department of Ophthalmology, Tsukazaki Hospital, Hyogo, JPN

**Keywords:** accumulation of copper, wilson`s disease, chronic lymphocytic leukemia, monoclonal gammopathy, hypercupremia, corneal opacity

## Abstract

Hypercupremia-induced corneal copper deposition secondary to monoclonal gammopathy is rare and shows a characteristic corneal opacity quite different from other causes of hypercupremia, such as Wilson's disease. This report describes a case of corneal copper deposition in a patient with monoclonal gammopathy associated with chronic lymphocytic leukemia. An 84-year-old man with slowly progressive corneal opacity was referred to our hospital. The corneal opacity was present at least five years ago. The patient's best-corrected visual acuity was 20/25 OU (in both eyes) at the initial visit to our hospital. Slit-lamp examination and anterior segment optical coherence tomography revealed bilateral brown-colored opacity localized to deep layers of the central cornea. *In vivo* confocal microscopy (IVCM) showed indistinct corneal stromal cells in the deep layer and endothelial cells. The possible differential diagnoses were corneal dystrophy and Wilson's disease, but the color, shape, or site of corneal opacity was inconsistent with the disease. As the patient had a history of chronic lymphocytic leukemia, which is often associated with monoclonal gammopathy, we suspected that the corneal opacity was copper deposition in association with the hematologic diseases. Laboratory examinations showed elevated serum copper and normal ceruloplasmin. Serum protein electrophoresis revealed significantly high IgG levels with depression of IgA, IgE, and IgM. These results supported our diagnosis. Followingly, we consulted the patient's attending hematologist, and the doctor initiated treatment for hypercupremia. In conclusion, hypercupremia secondary to monoclonal gammopathy should be considered a possible cause of central brown-colored corneal opacity.

## Introduction

Hypercupremia can lead to corneal copper deposition, typically associated with the peripheral Kaiser-Fleischer ring in Wilson's disease [1]. This report focuses on an unusual presentation of corneal copper deposition: bilateral brown-colored corneal opacity in the central cornea. The case involves a patient with chronic lymphocytic leukemia (CLL) and associated monoclonal gammopathy. Several reports have shown that monoclonal gammopathy can cause central copper deposits, but such cases are quite rare [2-7].

CLL, often linked to monoclonal gammopathy [8], is central to our exploration of this unique corneal manifestation. The rarity of this presentation prompts deeper investigation into the interplay between hypercupremia, monoclonal gammopathy, and ocular manifestations. By highlighting this atypical case, we aim to contribute to understanding ocular complications in hematologic disorders and help ophthalmologists diagnose the rare pathology.

## Case presentation

An 84-year-old Japanese man with slowly progressive corneal opacity was referred to Hiroshima University Hospital. The patient's corneal opacity was present at the initial visit to the former ophthalmologist five years ago. The patient's medical history included type two diabetes mellitus, hyperuricemia, cholangiocarcinoma after pancreatoduodenectomy, and chronic lymphocytic leukemia (CLL). The patient used to be treated with rituximab for CLL and has been in remission without any treatment for over nine years. The patient's family history was negative for any ocular disease.

**Figure 1:**
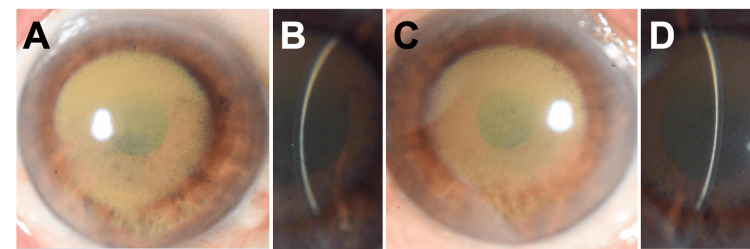
The slit-lamp photographs at the first visit to our hospital. Round and brown-colored opacity is located at the central cornea in both eyes: (A) the right eye and (C) the left eye. Slit-beam illumination detects the corneal opacity in the deep layer: (B) the right eye and (D) the left eye.

The patient's best-corrected visual acuity was 20/25 OU at the initial visit to our hospital. The intraocular pressure was 13 mmHg in the right eye (OD) and 14 mmHg in the left eye (OS). Slit-lamp examination showed round and brown-colored opacity at the central and deep cornea in both eyes (Figure 1). Pigmentation also appeared on the central iris. The dense corneal opacity made it difficult to visualize the details of the lens.

**Figure 2:**
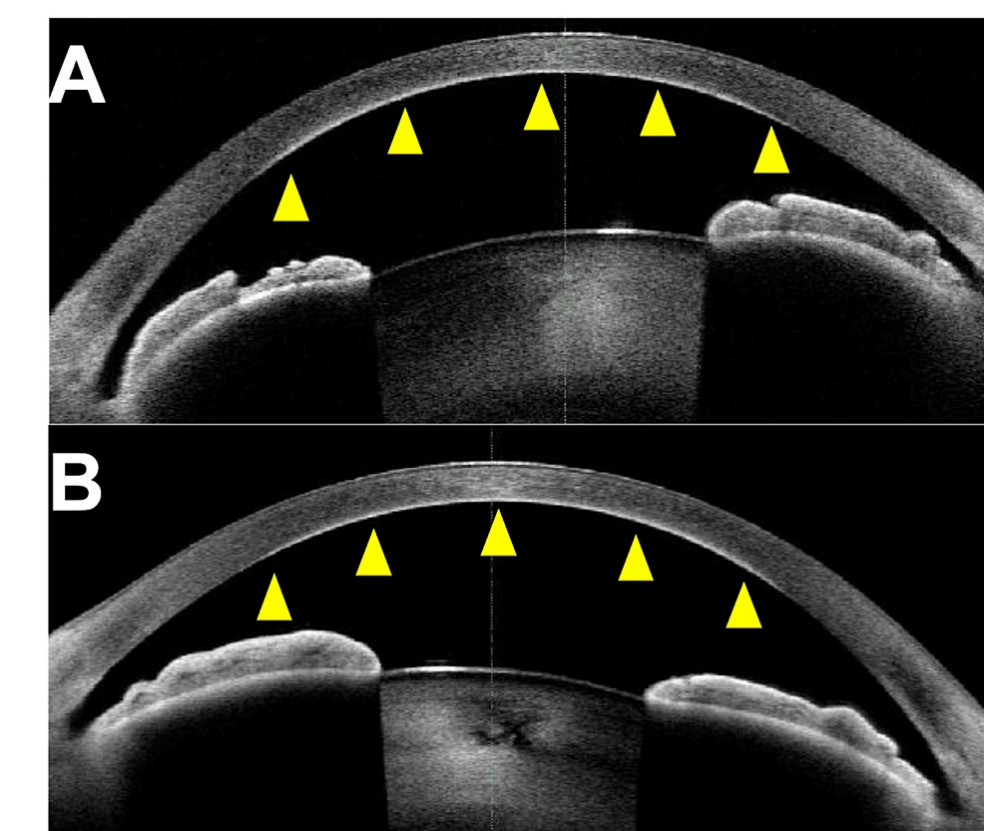
Anterior segment optical coherence tomography images. A hyperreflective line appears at the deep layer of the central cornea in both eyes: arrowheads; (A) the right eye and (B) the left eye.

Anterior segment optical coherence tomography detected hyperreflective materials at the deep layer of the cornea (Figure 2).

**Figure 3:**
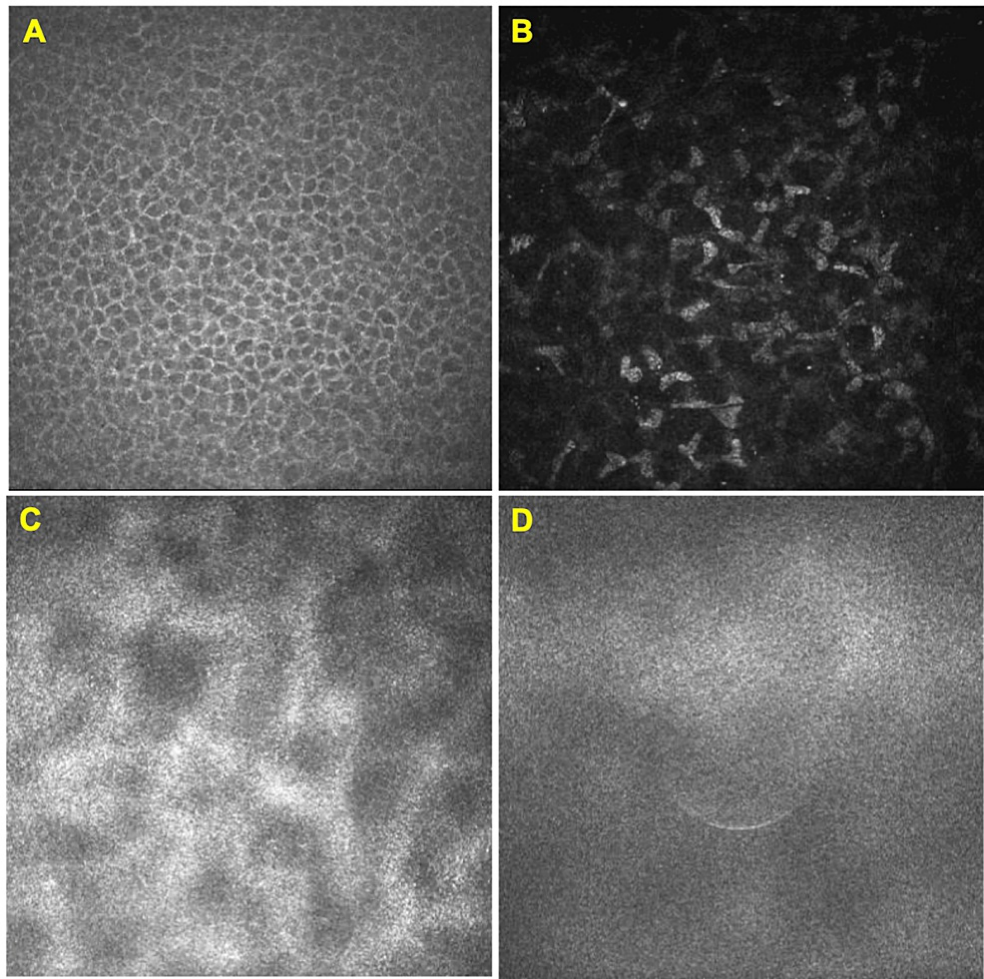
In vivo confocal microscopy images of the right eye. (A) No abnormal structure exists, such as inflammatory cells in the epithelial layer. (B) None of the stromal cells is activated in the shallow stromal layer. (C and D) The detailed structure in the deep stromal and endothelial layers is indistinct, although the stromal cells seem activated. No deposit was detected through all layers of the affected cornea.

In vivo confocal microscopy (IVCM) showed indistinct corneal stromal cells in the deep layer and endothelial cells. In contrast, epithelial and stromal cells in the shallow layer were normal and clearly captured. IVCM did not find any deposits (Figure 3).

Posterior segment ocular examination was unremarkable. Laboratory examinations showed elevated serum copper (630 μg/dL) and normal ceruloplasmin (20 μg/dL), alanine aminotransferase, and aspartate aminotransferase. Serum protein electrophoresis revealed a high immunoglobulin G (IgG) level of 3,172 mg/dL with depression of IgA, IgE, and IgM. The characteristic ocular findings and the results of systematic examinations suggested that the corneal opacity was copper deposition secondary to hypercupremia associated with IgG monoclonal gammopathy. Followingly, we consulted the patient with the attending hematologist and initiated the treatment of hypercupremia with zinc acetate dihydrate.

## Discussion

Bilateral brown-colored opacity in the central cornea is significantly characteristic of copper deposition secondary to hypercupremia associated with monoclonal gammopathy. However, this condition may not be well-recognized due to its low frequency. We could not make the correct diagnosis immediately in this case. We first considered the possibility of Schnyder corneal dystrophy (SCD). The shape and site of corneal opacity evoked SCD, but the corneal opacity of SCD appears in shallower layers than in our case and is not pigmented. Besides, phospholipids and cholesterol should be detected as spindle-shaped deposits by IVCM in SCD [8]. Another differential diagnosis was Wilson's disease. The brown-colored opacity at Descemet's membrane in our case was similar to Wilson's disease, but its site was the opposite. Besides, laboratory data of our patient, normal ceruloplasmin and normal hepatic function, were not consistent with Wilson's disease [1]. We also noted the patient's medical history of pancreatoduodenectomy as a possible cause of hypercupremia or corneal copper deposition, but there was no apparent relation between them in literature to the best of our knowledge. Finally, as monoclonal gammopathy is known to be associated with CLL [9], the patient's medical history allowed us to make the proper diagnosis in this case.

Shah et al. reviewed eight cases of corneal copper deposition secondary to hypercupremia associated with monoclonal gammopathy, including three multiple myeloma cases and five monoclonal gammopathies of unknown significance [4]. Whether the hematologic tumor is malignant or not, monoclonal gammopathy can cause hypercupremia. Bilateral brown-colored corneal opacity at the central Descemet's membrane was observed in all reviewed cases. Corneal transplant and cataract extraction were performed in some cases, revealing the presence of a pigmented band at the Descemet membrane. This was confirmed as copper deposition through Rhodanine staining and X-ray microanalysis [3,4]. Besides, although the detail of the lens surface was not visualized as satisfactory in our case due to its dense corneal opacity, some case reports showed that pigment also appeared on the lens capsule and was confirmed as a copper deposit pathologically [3,4].

Elevated IgG, which has a high affinity to plasma copper, is suggested to play an important role in the mechanism for why copper deposits accumulate at the central cornea in patients with monoclonal gammopathy [3]. While copper cannot penetrate the anterior chamber unless the blood-aqueous barrier breaks down, IgG can move into the anterior chamber [10] and thus may transfer plasma copper there. However, since excess copper from the bloodstream is believed to penetrate the anterior chamber and accumulate at the Descemet's membrane in Wilson's disease, the hypothesis may not safely explain why copper accumulates in different sites between monoclonal gammopathy and Wilson's disease [11]. Additionally, while acknowledging that excess IgG is a crucial factor in the characteristic corneal opacity observed in patients with monoclonal gammopathy, it should be noted that the immunoglobulin is present throughout all areas of the cornea, both centrally and peripherally, at similar levels [12]. Therefore, further investigation still seems necessary to comprehend the mechanism of the characteristic copper-deposition pattern in monoclonal gammopathy.

One obvious limitation of this case report is that we did not conduct a histopathological examination because the patient did not hope to receive surgical treatments. Nonetheless, given that the patient's corneal opacity was quite distinct and laboratory examinations were consistent with hypercupremia secondary to monoclonal gammopathy, we believe the lack of histopathological examination was not critical to diagnose.

## Conclusions

Hypercupremia secondary to monoclonal gammopathy should be considered a possible cause of bilateral brown-colored opacity in the central cornea. In this case, monoclonal gammopathy and hypercupremia had not been mentioned prior. Therefore, it is clinically meaningful that the ocular findings led to treating systematic diseases that would cause various systematic disorders.
